# The role of tumor necrosis factor-α and TNF-α receptors in cerebral arteries following cerebral ischemia in rat

**DOI:** 10.1186/1742-2094-8-107

**Published:** 2011-08-28

**Authors:** Aida Maddahi, Lars S Kruse, Qing-Wen Chen, Lars Edvinsson

**Affiliations:** 1 Department of Clinical Sciences, Division of Experimental Vascular Research, Lund University, Sweden; 2 Department of Clinical Experimental Research, Glostrup University Hospital, Glostrup Denmark

## Abstract

**Background:**

Tumour necrosis factor-α (TNF-α) is a pleiotropic pro-inflammatory cytokine, which is rapidly upregulated in the brain after injury. TNF-α acts by binding to its receptors, TNF-R1 (p55) and TNF-R2 (p75), on the cell surface. The aim of this study was first to investigate if there is altered expression of TNF-α and TNF-α receptors in cerebral artery walls following global or focal ischemia, and after organ culture. Secondly, we asked if the expression was regulated via activation of the MEK-ERK1/2 pathway.

**Methods:**

The hypothesis was tested *in vivo *after subarachnoid hemorrhage (SAH) and middle cerebral artery occlusion (MCAO), and *in vitro *by organ culture of isolated cerebral arteries. The localization and amount of TNF-α, TNF-α receptor 1 and 2 proteins were analysed by immunohistochemistry and western blot after 24 and 48 h of organ culture and at 48 h following SAH or MCAO. In addition, cerebral arteries were incubated for 24 or 48 h in the absence or presence of a B-Raf inhibitor (SB386023-b), a MEK- inhibitor (U0126) or an NF-κB inhibitor (IMD-0354), and protein expression evaluated.

**Results:**

Immunohistochemistry revealed enhanced expression of TNF-α, TNF-R1 and TNF-R2 in the walls of cerebral arteries at 48 h after MCAO and SAH compared with control. Co-localization studies showed that TNF-α, TNF-R1 and TNF-R2 were primarily localized to the cell membrane and the cytoplasm of the smooth muscle cells (SMC). There was, in addition, some expression of TNF-R2 in the endothelial cells. Immunohistochemistry and western blot analysis showed that these proteins were upregulated after 24 and 48 h in culture, and this upregulation reached an apparent maximum at 48 h of organ culture. Treatment with U0126 significantly reduced the enhanced SMC expression of TNF-α, TNF-R1 and TNF-R2 immunoreactivities after 24 and 48 h of organ culture. The Raf and NF-κB inhibitors significantly reduced organ culture induced TNF-α expression while they had minor effects on the TNF-α receptors.

**Conclusion:**

The present study shows that cerebral ischemia and organ culture induce expression of TNF-α and its receptors in the walls of cerebral arteries and that upregulation is transcriptionally regulated via the MEK/ERK pathway.

## Background

Stroke is a serious neurological disease and a leading cause of death and severe disability in the world [1]. There are two major kinds of stroke: ischemic stroke and hemorrhagic stroke. Both are associated with disruption of the blood flow to the brain with rapid depletion of cellular energy and glucose, resulting in ionic disturbances [2,3]. This initiates a complex process that includes release of excitatory neurotransmitters and activation of apoptotic pathways. Several investigators have shown that inflammation evolves within a few hours after cerebral ischemia. This inflammatory reaction involves accumulation of neutrophils, monocytes and leukocytes in the ischemic brain in animal models and in human focal stroke [3,4]. There is an early accumulation of neutrophils in the brain and transmigration of adhesion molecules that are associated with cytokine signaling. Stroke induces production and release of cytokines such as tumor necrosis factor-α (TNF-α), interleukin-1ß (IL-1ß), interleukin-6 (IL-6), and inducible nitric oxide synthase (iNOS), by a variety of activated cell types; endothelial cells, microglia, neurons, leukocytes platelets, monocytes, macrophages and fibroblasts [3,4]. We have found increased expression of iNOS and cytokines after middle cerebral artery occlusion (MCAO) [5] and after subarachnoid hemorrhage (SAH) [6] localized in smooth muscle cells of cerebral arteries and in the walls of associated intracerebral microvessels.

TNF-α is a pleiotropic cytokine produced by many cell types, and is involved in blood-brain barrier, inflammatory, thrombogenic, and vascular changes associated with brain injury [7]. TNF-α has been suggested to stimulate angiogenesis following ischemia through induced expression of angiogenesis-related genes [8,9]. It is known as a strong immunomediator and pro-inflammatory cytokine, which is rapidly upregulated in the brain after injury and is associated with necrosis or apoptosis [10]. TNF-α effects are mediated via two receptors, TNF-R1 (p55) and TNF-R2 (p75), on the cell surface [11]. TNF-R1 is expressed on all cell types and can be activated by both membrane-bound and soluble forms of TNF-α. This is a major signaling receptor for TNF-α. The TNF-R2 is expressed primarily on hemopoietic and endothelial cells, responds to the membrane-bound form of TNF-α, and mediates limited biological responses [11]. TNF-α and its receptors may activate the nuclear factor-κB (NF-κB) pathway, which in turn may inhibit TNF-α-induced cell death [12]. NF-κB is a pivotal transcriptional factor down-stream of MAPK and PKC pathways and its activation is essential for controlling the expression of several genes involved in inflammation and cell proliferation [13,14]. Increased TNF-α level has been observed in brain tissue, plasma and cerebrospinal fluid in Alzheimer's disease, multiple sclerosis and Parkinson's disease [15-17].

The present study aimed to address two questions: First, is the expression of TNF-α, TNF-R1 and TNF-R2 altered in cerebrovascular smooth muscle cells (SMCs) following MCAO, SAH and organ culture? Second, what intracellular signaling events are involved in regulating the expression of these molecules? This was examined by in vitro application of signal transduction blockers, such as the MEK/ERK1/2 inhibitor U0126, the B-Raf inhibitor SB3860-b, and the NF-κB inhibitor IMD-0354 [18,19]. The studies included an analysis of the levels of expression of TNF-α, TNF-R1 and TNF-R2 in cerebral arteries under the different experimental conditions by both immunofluorescence and western blot.

## Materials and methods

### Middle cerebral artery occlusion

Male Wistar-Hanover rats (Møllegaard Breeding Centre, Copenhagen, Denmark), weighing approximately 300-350 g (n = 6 per group), were obtained from Harlan Horst, the Netherlands. The animals were housed under controlled temperature and humidity with free access to water and food. The experimental procedures were approved by the Lund University Animal Ethics Committee (M43-07). Anaesthesia was induced using 4.5% halothane in N_2_O:O_2 _(70%:30%) and thereafter the animals were kept anaesthetized through a mask with 1.5% halothane during the operation. To confirm proper occlusion of the right MCA a laser-Doppler probe (Perimed, Järfälla, Sweden) was fixed on the skull (1 mm posterior to the bregma and 6 mm from the midline on the right side) to measure regional cortical blood flow. A polyethylene catheter was inserted into a tail artery to measure the mean arterial blood pressure, pH, pO_2_, pCO_2_, and plasma glucose. A rectal temperature probe connected to a homeothermal blanket was used to maintain body temperature at 37°C during the procedure. An intraluminal filament technique was used to induce transient MCAO, previously described in detail by Memezawa [20]. The resulting occlusion was visible by laser Doppler flowmetry as an abrupt 80-90% reduction of cerebral blood flow. Two hours after MCA occlusion, the rats were briefly reanesthetized to allow withdrawal of the filament to achieve reperfusion and normalization of flow. At 48 h post MCA occlusion, the rats were anesthetized and decapitated, the brains were removed and the MCAs were harvested. The right cerebral artery had been subjected to cerebral ischemia and the left served as a control (see below for details). The infarct volume, neurological score and physiological parameters were calculated and published in previous studies [21,22]. There were no significant differences in physiological parameters between the different treatment groups, such as blood pressure, blood gases, temperature, plasma glucose, and body weight. We observed an infarct volume (24.8 ± 2% of total cerebrum in the MCAO group) and evaluated the neurological score just before animal sacrifice (MCAO group, 4.0 ± 0.2 versus sham-operated animals with no visible defects resulting in a score of 0) [21,22].

### Rat subarachnoid hemorrhage model

Subarachnoid hemorrhage was induced by a model originally devised by Svendgaard et al [23] and described in detail by Prunell et al [24]. Male Sprague-Dawley rats (n = 6 per group) weighing approximately 350-400 g were anaesthetized using 5% halothane (Halocarbon Laboratories, River Edge, New Jersey) in a N_2_O:O_2 _mixture (ratio 30:70). All animal experiments were performed following the national laws and guidelines and were approved by the Danish Animal Experimentation Inspectorate and the Ethics Committee for Laboratory Animal Experiments at the University of Lund. The rats were intubated and artificially ventilated with inhalation of 0.5-1.5% halothane in a N_2_O:O_2 _mixture (ratio 70:30) during the surgical procedure. The depth of anaesthesia was carefully monitored and the respiration checked by regularly withdrawing arterial blood samples for blood gas analysis (Radiometer, Copenhagen, Denmark). A temperature probe was inserted into the rectum of each rat to record the body temperature, which was maintained at 37°C by a heating pad. An arterial catheter to measure blood pressure was placed in the tail artery, and a catheter to monitor intracranial pressure (ICP) was placed in the subarachnoid space under the subocciptal membrane. At either side of the skull, two laser-Doppler flow probes were placed over either hemisphere to measure cortical cerebral blood flow (CBF). Finally, a 27 G blunt canula with side hole was introduced 6.5 mm anterior to bregma in the midline at an angle of 30° to the vertical using a stereotactic frame. After 30 min of equilibration of the animal, 250 μl blood was withdrawn from the tail catheter and injected intracranially at a pressure equal to the mean arterial blood pressure (MABP) (80-100 mmHg). Subsequently, the rat was kept under anaesthesia for another 60 minutes to allow recovery from the cerebral insult after which catheters were removed and incisions closed. For a more detailed description, see previous studies [6,25]. During the recovery period, the rat was monitored regularly, and if it showed severe distress, the animal was euthanized (8% mortality). In addition, a series of sham-operated rats were prepared. They went through exactly the same procedure as described above except that no blood was injected intracisternally. The physiological parameters and cerebral blood flow have been reported before [6]. In that study, we observed no statistical difference in physiologic parameters among the groups (sham, SAH). As a result of the injection of blood, the cortical blood flow dropped in both hemispheres to 14 ± 5% of the resting flow and the intracranial pressure increased from 12 ± 2 to 121 ± 9 mmHg. These values normalised within 30 min [6]. There was a significant decrease in CBF as measured at 48 h in the SAH group (63 ± 2 mL per 100 g per minute; P < 0.05) as compared with the control group (140 ± 6 mL per 100 g per minute; P < 0.05) and animals from the SAH group showed a reduction in regional CBF in 16 of the 18 brain regions examined as compared with the control (sham) group [6]. Following the procedure described, harvesting of vessels was done at 48 h post SAH (see below for details).

### Harvesting cerebral arteries and brain tissue

After 48 hours of observation, MCAO, sham and SAH rats were anaesthetized using CO_2 _and decapitated. The brains were removed and immersed in ice-cold bicarbonate buffer solution. The right and left MCAs, and the basilar artery (BA) were dissected out using a dissection microscope, snap frozen, and stored at -80°C for immunohistochemistery.

### Tissue preparation and organ culture

A total of 111 Male Wistar Hannover rats (Møllegaard Breeding Center, Copenhagen, Denmark), weighing 350 to 420 g, were used for organ culture. The animals were anesthetized with CO_2 _and decapitated. The brains were quickly removed and chilled in ice-cold bicarbonate buffer solution. The BA, the right and left MCAs, and the circle of Willis were removed and dissected free from the brain and surrounding tissue under a dissection microscope. The artery segments were placed individually into wells of a 12-well plate with 2 ml serum-free Dulbecco's modified Eagle's medium (DMEM) [26]. Incubation was performed at 37°C in humidified 5% CO_2 _in air for 24 or 48 h in the presence or absence of the intracellular signal inhibitors. The arteries were transferred into new wells containing fresh medium every 24 h. After 24 or 48 h, the vessels were snap frozen and stored at -80°C for immunohistochemistery and western blot.

### Buffers, chemicals and drugs

The specific inhibitors used included: an IkB kinase 2 (IKK-2) inhibitor IMD-0354 (N-(3, 5-Bis-trifluoromethylphenyl)-5-chloro-2-hydroxybenzamide) (30 nM) [27], a specific MEK1/2 inhibitor U0126 (10 μM) and a specific B-Raf inhibitor SB386023-b (10 μM) [28]. IMD-0354 and U0126 were obtained from Sigma (St Louis, MI, U.S.A) and SB386023b was a generous gift from Dr. A. Parsons at GlaxoSmithKline (GSK), UK. All inhibitors were dissolved in dimethylsulfoxide (DMSO) and further diluted in saline solution to the final concentrations used in the experiments. The bicarbonate buffer solution was of the following composition (mM): 119 NaCl, 15 NaHCO_3_, 4.6 KCl, 1.2 MgCl_2_, 1.2 NaH_2_PO_4_, 1.5 CaCl_2 _and 5.6 glucose. Dulbecco's modified Eagle's medium (DMEM) contained L-glutamine (584 mg/L) and was supplemented with penicillin (100 U/ml) and streptomycin (100 μg/ml) (Gibco BRL, Paisley, UK).

### Immunohistochemistry

Cerebral arteries from MCAO, SAH, sham, fresh, culture (24 and 48 h) and culture + inhibitors were placed into Tissue TEK (Gibo, Invitrogen A/S, Taastrup, Denmark), frozen on dry ice and sectioned into 10-μm thick slices in a cryostat (Microm HM500 M; Thermo Scientific, Walldorf, Germany). Three sections were collected and placed on each microscope slide (Menzel, Branuschweig, Germany) Mounted sections were fixed for 10 minutes in ice-cold acetone (-20°C) and then rehydrated in phosphate buffer solution (PBS) containing 0.3% Triton X-100 for 15 min. The sections were permeabilized and blocked for 1 h in blocking solution containing PBS, 0.3% TritonX-100, 1% bovine serum albumin (BSA) and 5% normal donkey serum, and then incubated over night at 4°C with the following primary antibodies: rabbit polyclonal to TNF-α (Abcam, ab66579) diluted 1:500, rabbit polyclonal to TNF-α receptor 1 (Abcam, ab19139) diluted 1:1800, and rabbit polyclonal to TNF-α receptor 2 (Abcam, ab15563) diluted 1:50. All primary antibodies were diluted in PBS containing 0.3% Triton X-100, 1% BSA, and 2% normal donkey serum. Sections were subsequently incubated for 1 h at room temperature with secondary Cy™^2^-conjugated donkey anti-rabbit (711-165-152; Jackson ImmunoResearch, Europe Ltd., Suffolk, UK) diluted 1:200 in PBS containing 0.3% Triton X-100 and 1% BSA. Finally, the sections were washed with PBS and mounted with anti-fading Vectashield mounting medium (Vector Laboratories Inc., Burlingame, CA, USA). Immunoreactivity was visualized using an epifluorescence microscope (Nikon 80i; Tokyo, Japan) at the appropriate wavelengths and photographed with an attached Nikon DS-2Mv camera. The same procedure was used for the negative controls except that either the primary or the secondary antibody was omitted to evaluate autofluorescence and non-specific secondary antibody binding levels.

### Double immunofluorescence

Double immunofluorescence labeling was performed for TNF-α, TNF-R1 or TNF-R2 and smooth muscle actin, a selective smooth muscle cell marker. For the latter, a mouse anti-rat smooth muscle actin antibody (SC-53015; Santa Cruz Biotechnology, Inc, Santa Cruz, CA) was used at 1:200, diluted in PBS containing 0.3% Triton X-100, 1% BSA, and 2% normal donkey serum. The secondary antibodies were Cy™^2^- conjugated donkey anti-rabbit (Jackson ImmunoResearch, Europe Ltd. Suffolk, UK) diluted 1:200 and Texas Red-labeled donkey anti-mouse (Jackson ImmunoResearch, Europe Ltd. Suffolk, UK) diluted 1:300 in PBS containing 0.3% Triton X-100 and 1% BSA. The sections were mounted with Vectashield mounting medium with 4', 6-diamidino-2-phenylindole (DAPI) (Vector Laboratories Inc., Burlingame, CA, USA). The antibodies were detected at the appropriate wavelengths using a Nikon confocal microscope (EZ-cl, Germany).

### Western blotting

Cultured MCA, BA and circle of Willis (CW) vessels were homogenized in cell extract denaturing buffer (BioSource, Carlsbad, CA) containing phosphatase inhibitor and protease inhibitor cocktails (Sigma, St Louis, MI, U.S.A). Whole cell lysates were sonicated on ice for 2 min, centrifuged at 15 000 × g at 4°C for 30 min, and the supernatants were collected as protein samples. Protein concentrations were determined using standard protein assay reagents (Bio-Rad, Hercules, CA) and stored at -80°C awaiting immunoblot analysis. The protein homogenates were diluted 1:1 (v/v) with 2× sodium dodecyl sulfate (SDS) sample buffer (Bio-Rad). Protein samples (25-50 μg of total protein) were boiled for 10 min in SDS sample buffer and separated on 4-15% SDS Ready Gel Precast Gels (Bio-Rad, USA) for 120 min at 100 V and transferred to nitrocellulose membranes (Bio-Rad) at 100 V for 60 min. The membrane was then blocked for unspecific binding for 1 h at room temperature with PBS containing 0.1% Tween-20 (Sigma) and 5% non-fat dried milk, thereafter incubated overnight at 4°C with primary antibodies: rabbit polyclonal anti TNF-α, TNF-R1 and TNF-R2 (1:250 dilution; Abcam) or mouse polyclonal anti β-actin (1: 15000 dilution; A5441, Sigma), followed by incubation with horseradish peroxidase (HRP)-conjugated anti-rabbit IgG secondary antibodies (1:40000; GE Life sciences, Piscataway, NJ) for 1 h at room temperature. The labelled proteins were developed using the LumiSensor Chemiluminescent HRP Substrate kit (GenScript Corp., Piscataway, NJ). To detect multiple signals on a single membrane, the membrane was incubated in Restore Plus western blot stripping buffer for 5-15 min at room temperature (Pierce Biotechnology, Inc., Rockford, IL) between the various labelling procedures.

### Calculations and statistical analyses

Fluorescence intensity was measured using the ImageJ software http://rsb.info.nih.gov/ij/. Measurements were made in 4 to 6 different areas (located on the clock at 0, 3, 6 and 9 h) for each section and 6 sections from each rat were evaluated. Thereafter, the mean values of the intensity per measured area were used (from five different rats per group). The increased intensity for the SAH group was compared to the sham group and for the MCAO the two sides were compared. Results are expressed as mean values ± S.E.M (in arbitrary units of fluorescence intensity). For the arteries in culture, the mean values were presented as percentage fluorescence in the culture groups compared to the fresh group, where the fresh group was set to 100%. The investigator was blinded to the treatment group of each sample.

For western blot, cerebrovascular protein lysates from the different groups were compared. Cerebral arteries from two rats were pooled for each measurement (n = 8-10 rats in each group; fresh, 24 h incubation, 48 h incubation, DMSO + 48 h incubation and inhibitors + 48 h incubation). Three independent experiments were performed in duplicate. The membranes were visualized using a Fujifilm LAS-4000 Luminescent Image Analyzer (Stamford, CT), and band intensity was quantified using Image Gauge Version 4.0 (Fuji Photo Film Co., Ltd., Japan). The immunoblot optical density values were presented as percentage activity in the vehicle and treated groups compared to the fresh groups, where the fresh group was set to 100%.

Data are expressed as the mean ± standard error of the mean (S.E.M). Statistical analyses were performed using the nonparametric Kruskal-Wallis with Dunn's post hoc test for comparison between more than two groups and Mann-Whitney test for comparison between two groups. *P*-values less than 0.05 were considered significant; "n" refers to the number of rats.

## Results

### Middle cerebral artery occlusion

**Figure 1:**
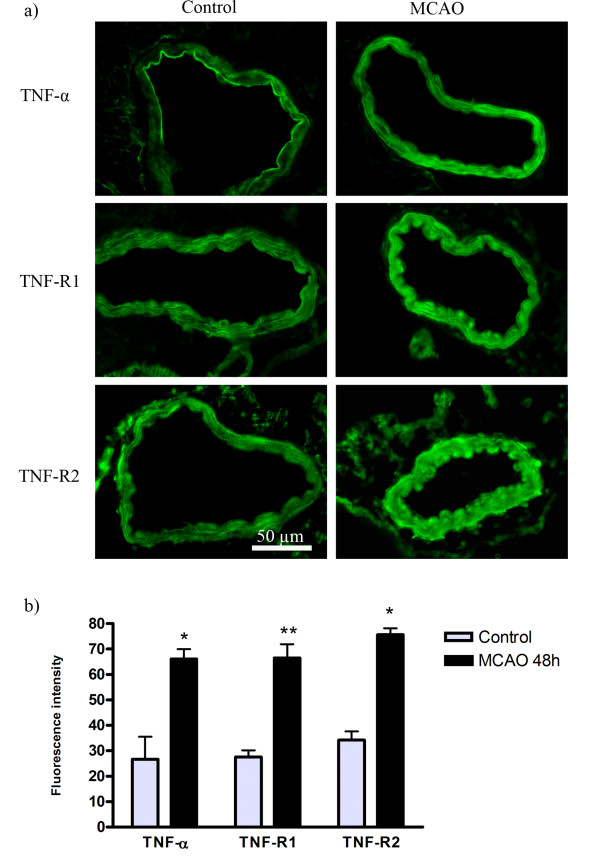
**Immunohistochemistry and image analysis of TNF-α, TNF-R1 and TNF-R2 protein levels in MCA following MCAO**. a) Typical examples that show the increased in TNF-α, TNF-R1 and TNF-R2 proteins in the artery SMCs wall of rat MCA following ischemic stroke in comparison to control animals. **b) **Bar graphs showing semi-quantification of fluorescence intensity for TNF-α, TNF-R1 and TNF-R2 in MCA. There were significant increases in the expression of these proteins in MCAO animals as compared to control. The results are expressed as mean values ± S.E.M. (in arbitrary units of fluorescence intensity, a.u.) *P < 0.05 and **P < 0.01, significant difference between control and MCAO.

The levels of TNF-α, TNF-R1 and TNF-R2 proteins expressed in the cerebral arteries after 48 h post MCAO for 2 h and reperfusion were investigated by immunofluorescence. Microscopic evaluation showed enhanced expression of TNF-α, TNF-R1 and TNF-R2 in smooth muscle cells in the MCAO group as compared with the control group (contralateral non-ischemic side) (Figure 1a). Measurements of fluorescence intensity confirmed the observations and revealed that the increase in protein expression was significant for all 3 markers: TNF-α (MCAO arteries 60 ± 5.2 a.u. compared to control arteries 25 ± 7.3 a.u., P < 0.05), TNF-R1 (MCAO arteries 66 ± 5 a.u. compared to control arteries 27 ± 2.3 a.u., P < 0.01) and TNF-R2 (MCAO arteries 65 ± 3.4 a.u. compared to control arteries 33 ± 4.3 a.u., P < 0.05) (Figure 1b).

### Subarachnoid hemorrhage

**Figure 2:**
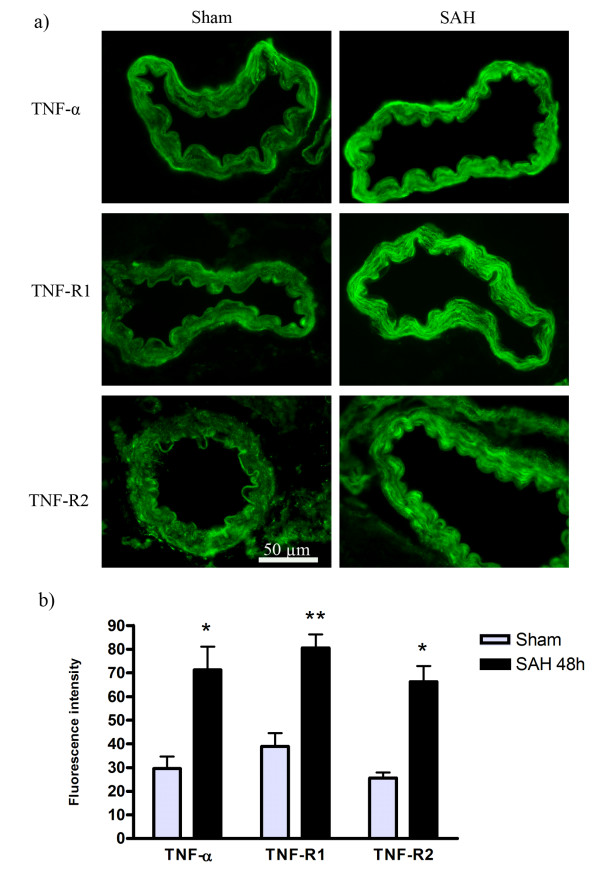
**Immunohistochemistry and image analysis of TNF-α, TNF-R1 and TNF-R2 protein levels in BA following SAH**. a) Photographs demonstrating the upregulation of TNF-α, TNF-R1 and TNF-R2 protein levels in the artery walls of rat basilar artery after experimental SAH in comparison to sham-operated animals. **b) **Bar graphs showing fluorescence intensity for TNF-α, TNF-R1 and TNF-R2 in BA. There were significant increases in the expression of these proteins in SAH animals as compared to sham animals The results are expressed as mean values ± S.E.M. *P < 0.05 and **P < 0.01, significant difference between sham and SAH.

Here we tested the hypothesis that TNF-α and its receptors might be upregulated in the artery wall at 48 h following SAH. Protein levels of TNF-α, TNF-R1 and TNF-R2 were investigated by immunofluorescence. Microscopic evaluation and fluorescence intensity measurements revealed that SAH resulted in a more intense immunoreactive signal corresponding to TNF-α (SAH arteries 72 ± 4 a.u. compared to sham arteries 30 ± 2.5 a.u., P < 0.05), TNF-R1 (SAH arteries 81 ± 5.6 a.u. compared to sham arteries 39 ± 5.6 a.u., P < 0.01) and TNF-R2 (SAH arteries 66 ± 6.5 a.u. compared to sham arteries 25.5 ± 2.3 a.u., P < 0.05) in smooth muscle cells of MCA and BA as compared to the sham group (Figures 2a and 2b).

### Organ culture

**Figure 3:**
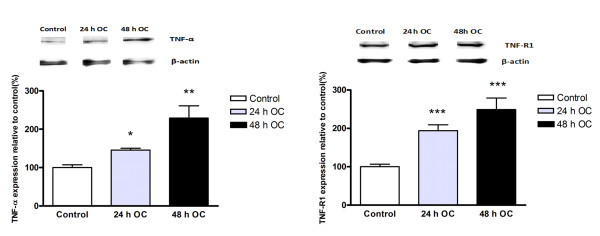
**Western blot showing TNF-α and TNF-R1 protein expression levels in the cerebral artery after 24 and 48 h organ culture (OC) as compared with fresh vessel (control)**. β-actin was used as a loading control. Data are expressed as mean ± S.E.M., n = 4. *P < 0.05, **P < 0.01 and *** *P *< 0.001.

**Figure 4:**
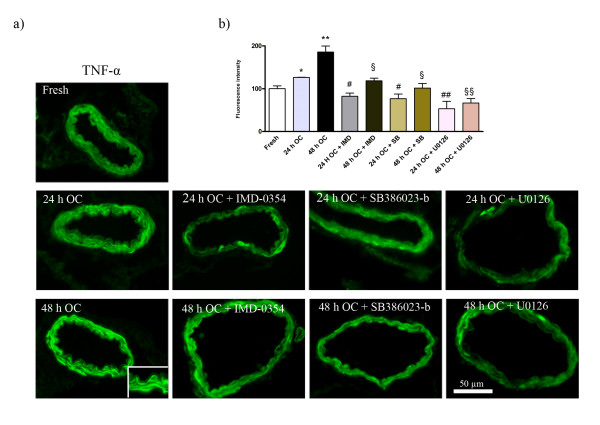
**Immunofluorescence staining for TNF-α; a) in the fresh, organ culture after 24 h incubation, organ culture after 48 h incubation, 24 h culture + treatment with IMD-0354, 48 h culture + treatment with IMD-0354, 24 h culture + treatment with SB386023-b, 48 h culture + treatment with SB386023-b, 24 h culture+ treatment with U0126 and 48 h culture + treatment with U0126**. There was a clear increase in TNF-α protein level in the smooth muscle cell layer after 24 and 48 h culture as compared to fresh vessels. All three inhibitors significantly prevented the enhanced expression of TNF-α protein level in culture for 24 and 48 h. **b) **Bar graph demonstrating the fluorescence intensity for TNF-α. The results are expressed as mean values ± S.E.M. *P < 0.05 and **P < 0.01, significant difference between fresh and culture after 24 and 48 h. ^§^P < 0.05 and ^§§^P < 0.01, significant difference between 48 h organ culture and treatment with 48 h culture + (IMD-0354, SB386023-b and U0126). ^#^P < 0.05 and ^##^P < 0.01, significant difference between 24 h organ culture and treatment with 24 h culture + (IMD-0354, SB386023-b and U0126).

**Figure 5:**
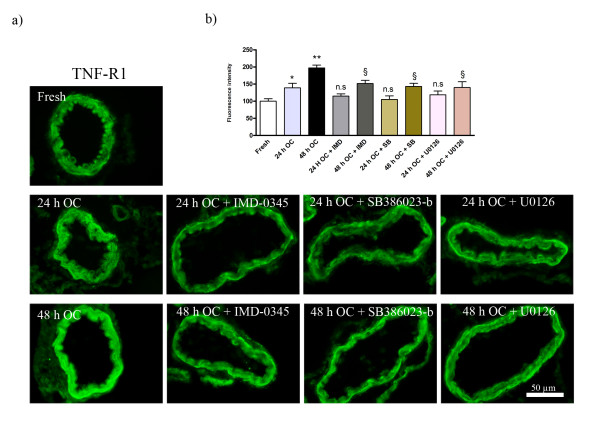
**Immunofluorescence staining for TNF-R1; a) in the fresh, organ culture after 24 h incubation, organ culture after 48 h incubation, 24 h culture + treatment with IMD-0354, 48 h culture + treatment with IMD-0354, 24 h culture + treatment with SB386023-b, 48 h culture + treatment with SB386023-b, 24 h culture+ treatment with U0126 and 48**. There was a marked increase in TNF-R1 protein level in the smooth muscle cell layer after 24 and 48 h culture as compared to fresh vessels. TNF-R1 was significantly decreased with all three inhibitors at 48 h of organ culture and reduced at 24 h. **b) **Bar graph demonstrating the fluorescence intensity for TNF-R1. The results are expressed as mean values ± S.E.M. *P < 0.05 and **P < 0.01, significant difference between fresh and culture after 24 and 48 h. ^§^P < 0.05 and ^§§^P < 0.01, significant difference between 48 h organ culture and treatment with 48 h culture + (IMD-0354, SB386023-b and U0126). ^#^P < 0.05 and ^##^P < 0.01, significant difference between 24 h organ culture and treatment with 24 h culture + (IMD-0354, SB386023-b and U0126). n.s. = not significant.

**Figure 6:**
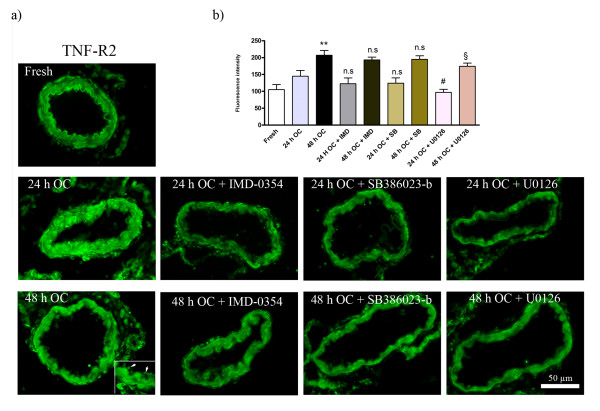
**Immunofluorescence staining for TNF-R2; a) in the fresh, organ culture after 24 h incubation, organ culture after 48 h incubation, 24 h culture + treatment with IMD-0354, 48 h culture + treatment with IMD-0354, 24 h culture + treatment with SB386023-b, 48 h culture + treatment with SB386023-b, 24 h culture+ treatment with U0126 and 48**. The expression of TNF-R2 increased in culture with a maximum in 48 h compare to fresh group. UO126 treatment significantly prevented this expression while, both IMD-0354 and SB386023-b slightly reduced the TNF-R2 protein level. **b) **Bar graph demonstrating the fluorescence intensity for TNF-R2. The results are expressed as mean values ± S.E.M. *P < 0.05 and **P < 0.01, significant difference between fresh and culture after 24 and 48 h. ^§^P < 0.05, significant difference between 48 h organ culture and treatment with 48 h culture + U0126. ^#^P < 0.05, significant difference between 24 h organ culture and treatment with 24 h culture + U0126. n.s. = not significant.

The relative expression levels of TNF-α, TNF-R1 and TNF-R2 proteins were investigated in cerebral arteries following 24 or 48 h of culture in the presence of U0126, SB-386023b, IMD-0354 or control solvent, and in fresh vessels (non-cultured). Western blot analysis revealed a significant increase in levels of TNF-α at 24 and 48 h as compared to fresh vessels (145.8 ± 4.8% and 229.2 ± 42.4%, respectively) and of TNF-R1 (193.3 ± 15.7% and 250.4 ± 30.4%, respectively) (Figure 3). Immunohistochemistry revealed enhanced expression of TNF-α, TNF-R1 and TNF-R2 both at 24 and at 48 h of culture, as compared with fresh vessels. The upregulation reached a maximum at 48 h (Figures 4, 5 and 6).

### Effect of NF-κB, B-Raf and MEK1/2 inhibitors on organ culture-induced upregulation of TNF-α and its receptors

In order to examine which intracellular signaling pathways that may be involved in the upregulation of TNF-α and its receptors, segments of cerebral arteries were cultured for 24 or 48 h with medium containing an IKK-2 inhibitor (IMD-0354, 30 nM), a B-Raf inhibitor (SB386023-b, 10 μM), the MEK1/2 inhibitor (U0126, 10 μM), or vehicle (same volume). Immunohistochemistry revealed enhanced expression of TNF-α, TNF-R1 and TNF-R2 in vehicle-treated samples, particularly at 48 h of culture (Figures 4, 5, 6 and table 1). This increase was prevented by treatment with U0126. In addition, there was a significant reduction in TNF-α immunoreactivity after treatment with IMD-0354. IMD-0345 significantly decreased also TNF-R1 immunoreactivity at 48 h incubation but not the immunoreactivity to TNF-R2. Treatment with the upstream B-Raf inhibitor (SB386023-b) abolished the upregulation of TNF-α and decreased the enhanced protein expression of TNF-R1 at 48 h (Figures 4, 5 and table 1).

**Table 1:** Levels of TNF-α, TNF-R1 and TNF-R2 proteins in the cerebral arteries in fresh vessels, in 24 and 48 h organ culture, with present and absences of IMD-0354, SB386023-b and U0126

	Fresh	24 h OC	48 h OC	IMD-0354 + 24 h OC	IMD-0354 + 48 h OC	SB386023-b + 24 h OC	SB386023-b + 48 h OC	U0126 + 24 h OC	U0126 + 48 h OC
TNF-α	100 ± 6	126 ± 1*	186 ± 10**	83 ± 7^#^	119 ± 7^§^	77 ± 10^#^	101 ± 9^§^	53 ± 8^##^	67 ± 9^§§^
TNF-R1	100 ± 7	139 ± 11*	197 ± 8**	115 ± 6	151 ± 8^§^	105 ± 6	143 ± 8^§^	118 ± 11	140 ± 6^§^
TNF-R2	100 ± 9	145 ± 15*	207 ± 14**	122 ± 7	193 ± 8	125 ± 7	194 ± 10	98 ± 8^#^	170 ± 9^§^

Values are expressed as percentage of fresh and given as mean values ± S.E.M, n = 5.

*P < 0.05 and **P < 0.01, significant difference between fresh and culture after 24 and 48 h.

^§^*P *< 0.05 and ^§§^*P *< 0.01, significant difference between 48 h organ culture and treatment with 48 h culture + (IMD-0354, SB386023-b and U0126).

^#^*P *< 0.05 and ^##^*P *< 0.01, significant difference between 24 h organ culture and treatment with 24 h culture + (IMD-0354, SB386023-b and U0126).

**Figure 7:**
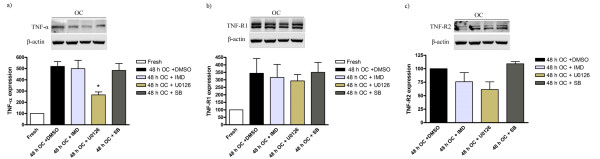
**Western blot of TNF-α, TNF-R1 and TNF-R2 protein levels from rat cerebral arteries; after 0 h (fresh), 48 h organ culture (OC) + DMSO, 48 h OC + (IMD-0354, SB386023-b or U0126) together with β-actin as loading control are shown**. UO126 treatment significantly decreased the increase protein level of TNF-α after 48 h culture (a). Data are presented as the TNF-α, TNF-R1 and TNF-R2/β-actin mean optical density ratio relative to fresh (a-b) or vehicle (48 h OC + DMSO) (c). Data are presented as mean S.E.M. *P < 0.05.

Western blot experiments using lysates from vessels incubated for 48 h with the inhibitors provided partial support for the immunohistochemistry results (Figure 7). A significant reduction in TNFα-like immunoreactivity was observed after treatment with U0126 as compared with DMSO + 48 h culture (Figure 7a). The MEK inhibitor showed a tendency to reduction (but not significant) of the increase in TNF-R1 after 48 h organ culture (Figure 7b) and for TNF-R2 (Figure 7c). We only studied TNF-R2 protein expression using western blot analysis after 48 h of organ culture and since there was no clear signal seen in fresh group this shows enhanced expression (Figure 7c). The other two inhibitors showed no significant variations in expression of TNFα and its receptors (Figure 7).

### Double immunofluorescence

**Figure 8:**
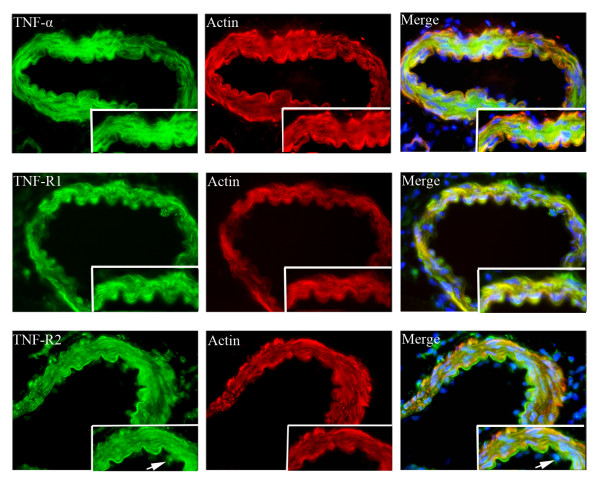
**Double-immunofluorescence staining for TNF-α, TNF-R1 and TNF-R2 (green) and smooth muscle actin (SMC) (red); triple staining with DAPI (which labels the cell nuclei; blue) of the cerebral arteries**. The enhanced expression of TNF-α and TNF-R1 was located in the cytoplasm and cell membrane of the smooth muscle cells (yellow color in merge). The TNF-R2 immunoreactivity was located in both SMC and endothelial layer (white arrows) (merge).

TNF-α, TNF-R1 and TNF-R2 proteins were localized mostly in the cytoplasm and cell membrane of SMCs in the medial layer of the cerebral artery (studied by co-localization with actin in the smooth muscle cells). Additionally, a weak expression of TNF-R2 was observed in the endothelial cell layer (co-localization with DAPI, which labels the cell nuclei) (Figure 8).

## Discussion

This study is the first to show that cerebral arteries show similar protein expression profiles with respect to TNF-α and its receptors in two *in vivo *cerebral ischemic models and during *in vitro *organ culture (used as a stress model). The upregulation observed here was confirmed by western blot quantification. In support, quantitative real time-PCR has demonstrated enhanced expression of TNF-α mRNA in cerebral vessels at 24 h after SAH, MCAO, organ culture, and in a time study between 1 to 48 h following SAH [29,30]. An upregulation is observed in the smooth muscle cells of cerebral arteries, which is regulated by the intracellular MEK-ERK pathway. The double immunofluorescence analysis demonstrated that TNF-α and TNF-R1 were primarily located in the cytoplasm and in the cell membrane of the SMCs, while the TNF-R2 immunoreactivity was located in cell membranes of both SMC and endothelial cells. The present study also showed that organ culture for 24 and 48 h induces an increased protein expression of TNF-α, TNF-R1 and TNF-R2 in a time-dependent manner. We observed enhanced expression of TNF-α and its receptors in cerebral microvessels following experimental MCAO and SAH (data not shown), which is in agreement with a previous report [5].

Several studies have recognized that inflammation is a key element in the pathophysiology and outcome after stroke [31]. Cytokines are polypeptides, generally associated with inflammation, immune activation and cell differentiation. TNF-α is a 17-Kd polypeptide cytokine that may affect growth, differentiation, cell proliferation, immunomodulation, survival, and the function of a variety of cells including those of the immune system, microglia, astrocytes and SMCs [4,11,32-34]. These cellular responses are mediated through two distinct TNF-α receptors: TNF-R1 is expressed on all cell types, whereas TNF-R2 is expressed only on cells of the immune system and on endothelial cells [35].

TNF-α, TNF-R1 and TNF-R2 mRNA levels have been shown to increase in the brain after both permanent and transient MCAO in rat and mouse [10,36,37], and in neuroretina and retinal arteries following ischemia in pig and mouse [12,38]. In closed head injury, the mRNA and functional activity (cytotoxicity) of TNF-α are increased [39] and increased TNF-α protein levels have been noted by western blot in the brain after stroke [40]. Thus, there is a correlation between TNF-α and brain damage.

Several investigators have suggested a role of the MAPK-MEK-ERK pathway in the regulation of TNF-α following cerebral ischemia. Studies have shown that TNF-α can increase the permeability of the blood-brain barrier (BBB) via activation of the ERK1/2 pathway, increase the expression of TNF-R1 and TNF-R2, and that treatment with U0126 inactivates this signaling pathway and decreases the expression of the TNF receptors [41,42]. Our findings with the MEK1/2 inhibitor are in concert with this.

Binding of TNF-α to its cell surface receptors results in activation of mitogen activated protein kinase (MAPK) which may lead to activation of e.g. two transcription factors, Activation Protein-1 (AP-1) and NF-κB [43]. NF-κB regulates expression of numerous components of the immune system, which includes pro-inflammatory cytokines, chemokines, adhesion molecules and inducible enzymes such as inducible nitric oxide synthase and cycloxygenase-2. Dysregulation of this signaling may result in inflammatory and autoimmune diseases [44]. NF-κB proteins are predominantly located in the cytoplasm, associating with members of the inhibitory IκB family such as IκB-α, IκB-β, IκB-ε. IκB proteins are believed to sequester NF-κB in the cytoplasm by masking its nuclear localization sequences. Thus, activation of NF-κB depends on degradation and phosphorylation of IκB [45].

In this study we have shown for the first time that *in vitro *organ culture of isolated cerebral arteries and *in vivo *ischemia models (MCAO and SAH) result in upregulation of TNF-α and TNF-α receptors after 48 h in cerebral vessels walls. Previous studies have revealed that organ culture of cerebral arteries results in upregulation of inflammatory factors such as cytokines and matrix metalloproteinases (MMPs) after 24 h in a way similar to that seen in ischemia models [29]. In addition, we have observed that after 24 h organ culture [28] and in experimental MCAO and SAH at 24 h [29] there is activation of MAPK cell signaling. We hypothesize that one major factor behind this is the change in shear stress which is caused by the rise in intracranial pressure and the reduction in wall tension in SAH or MCAO, and the removal of the intraluminal pressure during the organ culture procedure. We therefore suggest that organ culture can be used as a method to study mechanisms involved in enhanced expression of TNF-α and its receptors in cerebral arteries that occur following cerebral ischemia.

On the other hand the mechanical stress has been reported to activate MAPKs [46] and this event result in the activation of ERK1/2, P38 and JNK signal transducers. In addition, in a previous study [47] we reported in a western blot time study of global ischemia that there was early activation of the ERK1/2 pathway already within one hour while there was no activation of c-jun N-terminal kinase (JNK) or p38 at time points before 24 hours. Therefore, in order to elucidate the role of the intracellular MEK/ERK-NF-κB pathway in relation to upregulation of TNF-α and its receptors, we cultured cerebral artery segments for 24 or 48 h in the presence of SB386023-b or U0126 (10 μM), which block the upstream ERK1/2 signaling, and with IMD-0354 (30 nM), which blocks the downstream transcription factor NF-κB. The specificity of U0126, IMD-0354 and SB386023-b, in the doses used have been examined and reported in previous studies [18,19 and 48]. We observed that the enhanced expression of TNF-α was significantly reduced by treatment with each of these inhibitors in the immunohistochemistry part and this was confirmed for U0126 for the western blot. This is consistent with earlier work *in vivo *in which we showed that (i) SB386023-b following experimental SAH [6], and (ii) U0126 after MCAO [5] prevented enhanced cytokine expressions (TNF-α, IL-1β and IL-6). NF-κB is also activated early during organ culture (starting at 1 h) in rat mesenteric arteries [27]. The specific IKK-2 inhibitor (IMD-0354) has been shown to inhibit NF-κB activation by enhancing the stability of the IκB-NF-κB complex [49], thereby preventing the enhanced expression of TNF-α and its receptors in vascular SMC.

In the present study, by immunohistochemistry we report that all three inhibitors decreased expression of TNF-R1 protein at 48 h incubation. In contrast, the increased expression of TNF-R2 was significantly prevented only by U0126 treatment. The western blot experiments provided partial support for this by a tendency for inhibition of expression by U0126. One possible explanation for the difference might be that TNF-R1 contains a death domain in its cytoplasmic region whereas TNF-R2 lacks this [50]. Activation of TNF-R1 may lead to activation of the death domain, which activates the Ras and Raf kinases and thereafter phosphorylated pERK1/2 promotes activation of NF-κB by degradation of IκB. Therefore, blockade of phosphorylation and activation of this pathway can potentially inhibit the expression of TNF-R1, which correlates with suppression and inhibition of TNF-α expression [51]. In contrast, TNF-R2 can also activate NF-κB by a non-classical pathway, which is independent of degradation of IκB [42,52,53]. Administration of U0126 has been shown to reduce ischemic brain injury via inhibition of phosphorylated-MEK1/2 and phosphorylated- ERK1/2 expression, and prevents elevation of downstream transcription factors such as ELK-1, NF-κB and AP-1 phosphorylation [54].

## Conclusion

Our results show that cerebral ischemia (MCAO, SAH) and organ culture are associated with enhanced TNF-α, TNF-R1 and TNF-R2 protein levels in the wall of cerebral arteries. In addition, treatment with inhibitors of the MEK/ERK signaling pathway and NF-κB transcription factor prevent these enhanced expressions to various extents. The results suggest that inhibition of TNF signaling pathways by inhibition of the intracellular MAPK ERK1/2 pathway may constitute interesting targets, whereby inflammatory processes elicited following stroke may be prevented or attenuated.

## Competing interests

The authors declare that they have no competing interests.

## Authors' contributions

AM carried out the main part of the experiments, participated in the design, statistical analysis and writing of the manuscript. QC and LSK performed the western blot experiments and participated in writing of the final manuscript. LE conceived the study, directed the work, and drafted the manuscript. All authors have read and approved the final manuscript
